# Supplementary material for: A second generation leishmanization vaccine with a markerless attenuated Leishmania major strain using CRISPR gene editing
Source: Nat Commun. 2020 Jul 10;11:3461. doi: 10.1038/s41467-020-17154-z (PMC7351751; doi:10.1038/s41467-020-17154-z)
Supplement: Supplementary file 1 — Supplementary Information [file 41467_2020_17154_MOESM1_ESM.pdf]

SUPPLEMENTARY FIGURE 1

**A**

CTAGGCGATCGAGTCTTTGGCAGATGGCCGTTAGGGCGGTCAGTCTAAGTGGATGAGGATGTATTATGTTTTATAGTGAGGTTGAGGGGTGGCAGAAG ← F2
CGGAGGTGGAAGGAGACGAGCTGCCGATTGCGCAACTATCAGCACTGGTATCTACTGGGGGTTGTAGTGTATCGGGCCTAATCCATCGATGGGTGGCG
TAGCTGCTTCTGCAGTCGCCGTTGGCACGCGCTCTCAAGCAGTGAGGGAAGCCACTCTACATATCTAGGGCGCCCGCCTTTTCCGTTTCGCGGAAGTG
TTCTCATGCTCTATTTCGTGCTTCTCGCAATCCTTCTGCTTGGCCTGTTACCGGGGCTTACCGCGGCATACGACGGCTTCGTTGTCGGTGCATAATGC ← gRNAa
ACCCGCGCGTCGTGGAGCCACTCACAGTCATTTCCGTGTGTGCCCTGCATTCATCGACAGCATTCTGCATACAGCGTGAACGCGTCTCATAGAGCATA
TTTCCTTTATCGCGTTTTCGTTTGCACTGCCGCGATTTCTTTTCGTGGGTCGCATCAATCAACCATGGCTGCGCTTACGGATGAGCAGATTCGCGAGG
CCTTCAACCTCTTCGACGCCGACGGCTCTGGGGCTATCGACGCGGAGGAGATGGCGCTAGCGATGAAGGGTCTCGGCTTCGGTGACCTGTCGCGCGAC
GAGGTGGAGCGCATTATCCGCTCCATGCACACAGACTCCAACGGCCTGGTGGCGTACGGCGAGTTTGAAGCCATGGTCAAGTCGCGCATGGCGCAGAA ← Centrin ORF
GGACTCGCCGGAGGAGATCCTAAAGGCCTTTCAGCTCTTCGACCTCGATAAGAAAGGAAAAATCTCCTTTGCGAACTTGAAGGAGGTTGCGAAACTGC ← F1
TGGGTGAGAACCCCGGCGACGATGTGCTGAAGGAGATGATCGCCGAGGCCGATGAGGACGGTGATGGCGAGGTTTCCTTTGAGGAGTTCAAGAGCGTG
ATGCTGCAGATGCGTGGAAAGTAGAGGACGGCGCAGGAAGCGCTTGCGCCCTTCGCCTTCACCGCGCAGAGGAGATGCGATGGTCAAGGCAGTGAGGA
AAAGGCCGATACCGATCAAGAAATTCAAAAGTTCATTGTCATCAAGCGAAGGGGGCTAGTGGCCGAGGGCTCCGGAGTGTCACCGGAGTCACCTCGCA
CCGCTTGACCTCTGTCTCTCTCTCTCTCTCTCTCCTTGTCGCTTTACCTGCCCCACCTTTTGTGTTTTCGTCTGATTCAACGTTCTCAATAGGTGGTG
CTGTTGGTGATGATGATGATCGCGGTGTGTGCGTGTGCGTGCGTGCGCTTCCTTTCCCATCATCATCATCATCTGCCTTCACTTTGATTTCTCTTTCC
TTTGCGTAGGTTCGCGCCGTTCGGTTTTGGGCAGATCTTTTCTTTCGCTTTTGTATGGTTGTGCTGTCCGTTTCTATTTATGACTTCTTCGTGATTGT ← R1
ACACCCGGGGCCGTTGTCGTGCGTGGCGGCGCTGTGGTGTGTTGTCTGGGCTGGGTGGCTCTGTCCGTTGCTTTCCCTCTCAACGGATGATAGTGCGC
GTGTGCGTTCCTCTTCCTTCCTTTATCGTTTTGATCGCCGACAAGCTCGCTCAGTGGAACTCGGATAGTGTCGGAGAAGAGCAGGGCGAAGCCCCCCG ← gRNAb
CTCTGTAACGGCACCCTTTCTCACTCGTTCCGGCGTTCCACGGAGCAGTCGATAGATGAACACGGCGGGAAAGTTGGGAAGGCGGGGAGACGCTGAAT
GAACAGGGAAGGAATAGATGTCGTGGCTGAAAAACCAACGGAAACAAGCGAATCACGAT ← R2

**B**

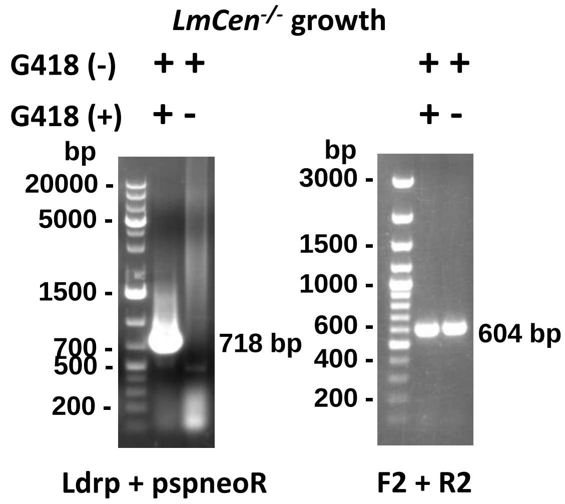


**C**

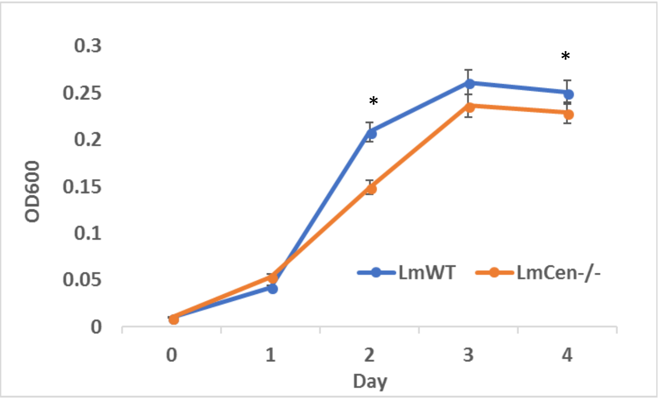


**D**

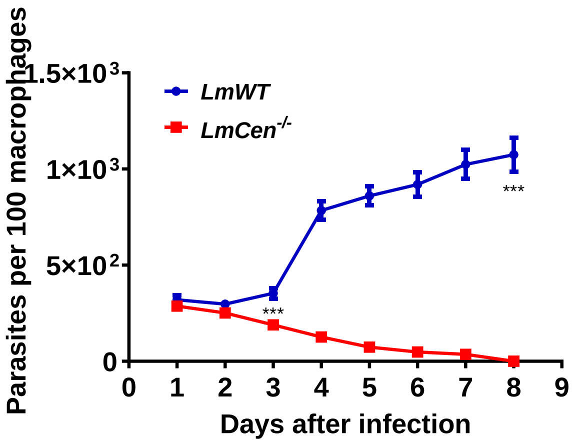

**Supplementary Figure 1**

**Characterization of *LmCen*$^{-/-}$ mutant parasite:** (**A**) *Leishmania major centrin* gene (LmjF.22.1410) and its flanking sequences. The CRISPR gRNAa and gRNAb targeting sites (Green) in the 5' and 3' *centrin* gene flanking sequences and PCR primers (Blue and underlined) used to confirm deletion of the *centrin* gene are indicated at the right. (**B**) Loss of antibiotic resistance CRISPR pLdCN plasmid in *LmCen-/-* cells after culture in G418 free medium for one month. The genomic DNA extracted from *LmCen-/-* cells which were still able or not able to grow in G418 containing medium were subject to PCR analysis with the CRISPR plasmid specific primers (LdrP, 5' GTGTGAGTTATGAGGTCTGCGA and pspneoR, 5' CTTGTTCAAGCTTGCGAATTCGAGC). No CRISPR plasmid specific PCR band was detected in the *LmCen-/-* cells which had lost the ability to grow in G418 containing culture medium, and the 604 bp F2+R2 band from the genome derived sequence could be detected in both G418 resistant and sensitive LmCen-/- cells. (**C**) *LmCen-/-* cells grow slower than wild-type *L. major* (*LmWT*) in promastigotes culture. *L. major* promastigotes were inoculated in a 96 well plate at the concentration of $10^6$ per ml, 120 ul per well and 5 wells per sample. The promastigotes growth was monitored by measuring the optical density in these wells at the wavelength of 600 nm (OD600) during the following 4 days. The data shown are the mean +/- SEM. Note, the cell density differences between *LmWT* and *LmCen-/-* cells at day 2 and 4 post inoculation are statistically significant (P=0.018 at day 2; 0.22 at day 3 and 0.026 at day 4; *p<0.05). Unpaired two tailed Student's t test was used to calculate statistical significance. This is the representative data of three independent experiments. (**D**) Human macrophages differentiated from monocytes were infected with stationary phase promastigote parasites from *LmWT* and *LmCen*$^{-/-}$ for six hours (10:1 parasite-to-macrophage ratio). The number of amastigotes in these cultures was determined over 8 days by microscopic observation of Diff-quik reagent stained slides. The data are expressed as the number of amastigotes per 100 macrophages. Data is representative of two independent experiments. Unpaired two tailed Student's t-test with no adjustments was used to calculate statistical significance. Error bars indicate the mean +/- standard deviation (*** p<0.001).

SUPPLEMENTARY FIGURE 2

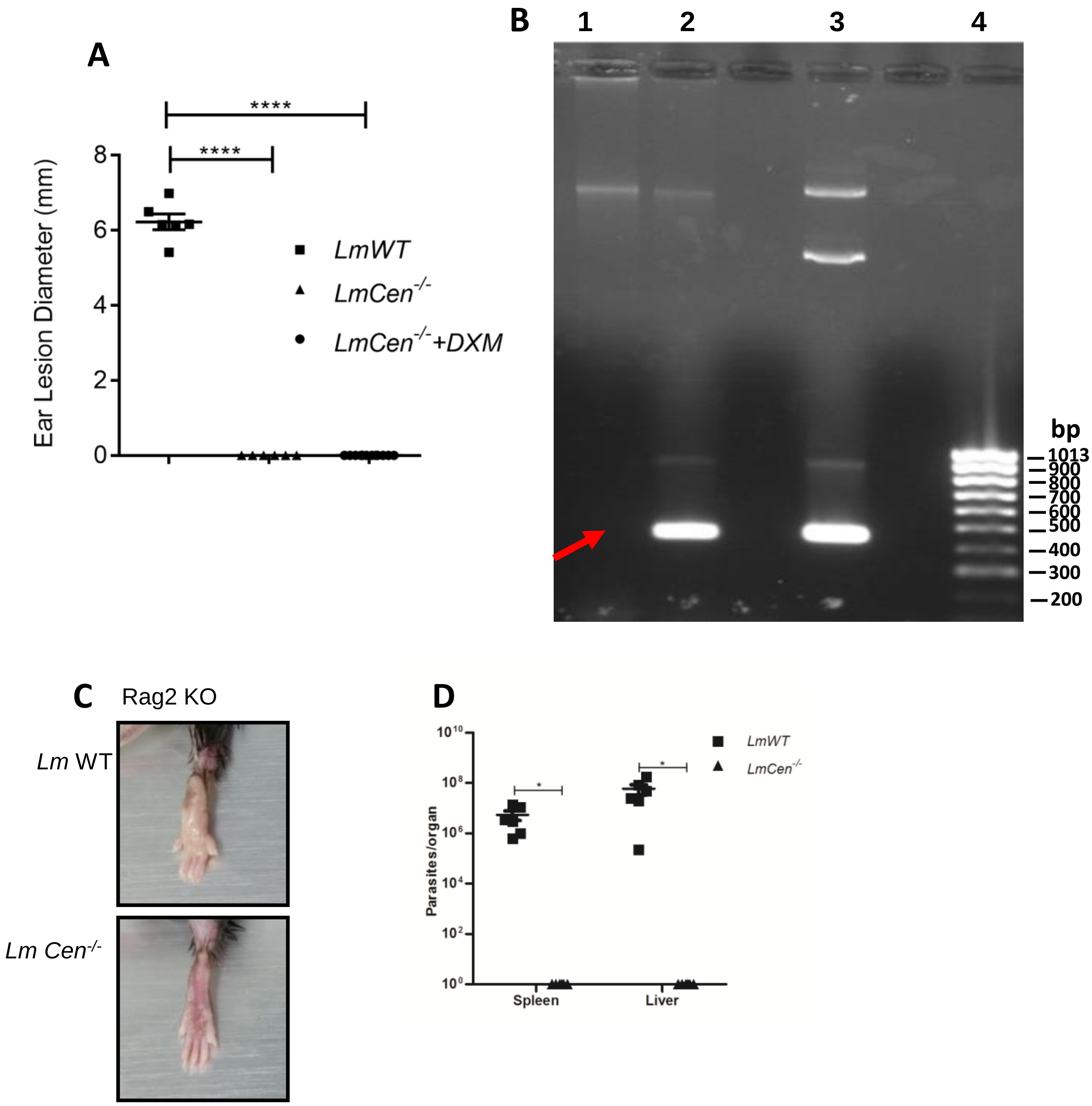

**SUPPLEMENTARY FIGURE 2:**

**Safety and non-pathogenicity. (A**) Ear lesion diameters was measured after 4 weeks of Dexamethasone (DXM) treatment (total 15 weeks post parasite infection) in *LmWT (n=6) and LmCen-/- (n=6) and LmCen-/-+DXM (n=12)* immunized mice. Results are mean ± SEM, of 1 ear, 6-12 mice per group. Data pooled from two independent experiments. Statistical analysis was performed by unpaired two-tailed t-test (****$p<0.0001$). (**B**) 1% Agarose gel electrophoresis results for the characterization of *LmCen*$^{-/-}$ parasites isolated from *LmCen*$^{-/-}$ plus DXM treated group using *L. major centrin* gene specific primers. Lane-1, PCR results from the genomic DNA of parasites isolated from *LmCen*$^{-/-}$ plus DXM treated group, Lane-2, PCR results from the genomic DNA of parasites isolated from *LmWT* group*,* Lane-3, PCR results from the plasmid DNA containing *centrin* gene as a positive control . Lane 4 , 100bp DNA ladder (Bioline) from 200bp to 1013bp respectively. Red arrow indicates the absence of main product bands (*centrin* gene) of 450 bp in Lane-1.Gel picture from one of two independent experiments (**C**) Representative photographs of footpad of Rag2 KO mice at 15 weeks post subcutaneous infection with $1 \times 10^7$ *LmCen*$^{-/-}$ (n=6) or *LmWT* (n=6) . Experiment performed only one time. (**D**) Parasite burden in spleen and liver of Rag2 KO mice at 15 weeks post subcutaneous infection with $1 \times 10^7$ of *LmCen*$^{-/-}$ (n=6) or *LmWT* (n=6) into footpad*.* Results are mean ± SEM. Data are representative of one experiment. Statistical analysis was performed by unpaired two-tailed t-test (*$p<0.04$)

SUPPLMENTARY FIGURE 3 :

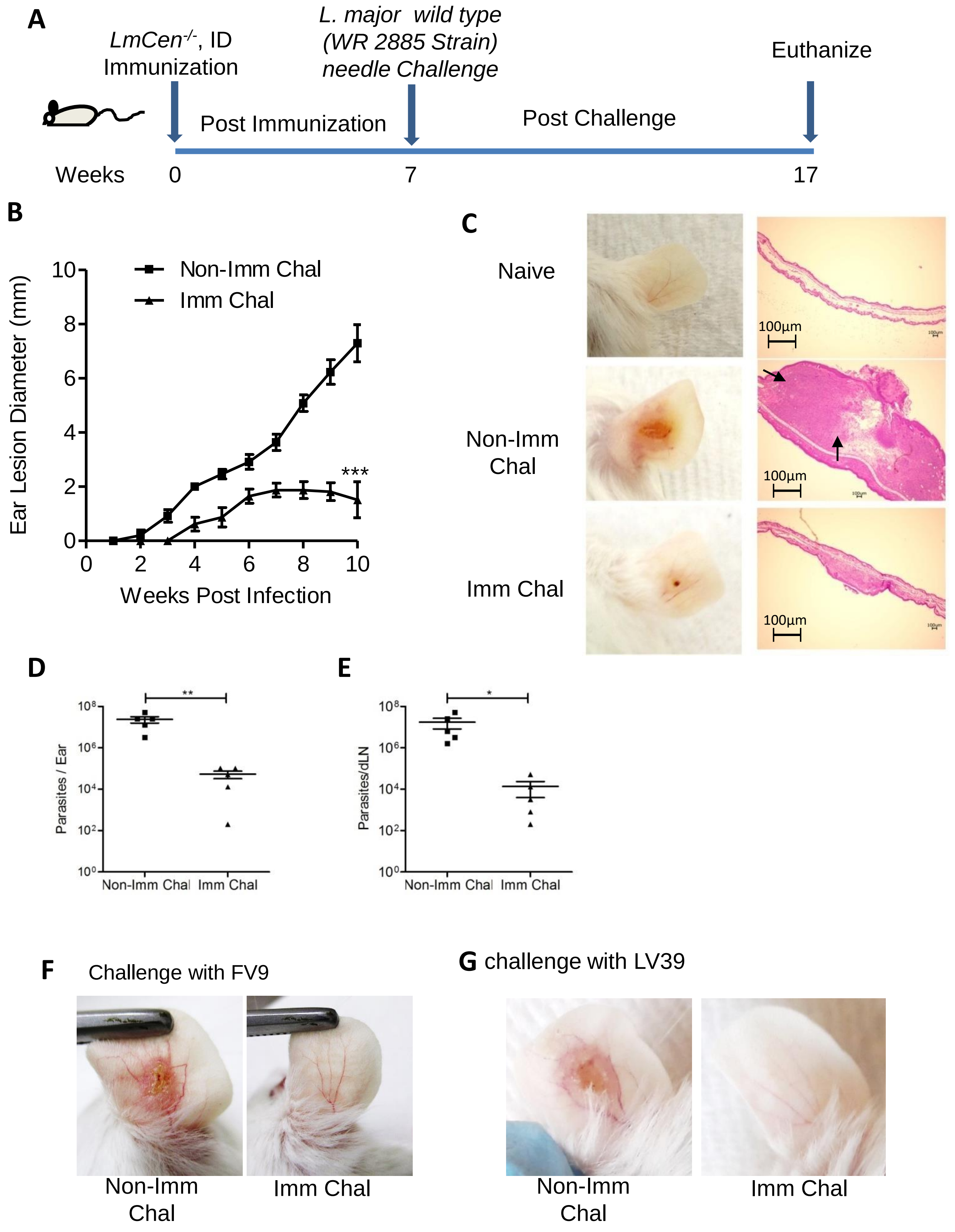

**SUPPLEMENTARY FIGURE 3**

**Protective efficacy of *LmCen$^{-/-}$* parasites against virulent *L. major* needle challenge in BALB/c mice.** (**A**) Schematic representation of the needle challenge procedure. (**B**) Ear lesion size were measured weekly for both *LmCen$^{-/-}$* immunized (Imm Chal) (n=5) and non-immunized (Non-Imm Chal) (n=5) mice after intradermal challenge with *LmWT* parasites. Results are represented as SEM, of 1 ear, 5 mice per group. Data are representative of one experiment. Statistical analysis was performed by unpaired one-tailed t-test (***p-0.0001). (**C**) Photographs (left panel) & histology (H&E stained, right panel) of representative challenged ear of *LmCen$^{-/-}$* immunized (Imm Chal) & non immunized (Non-Imm Chal) mice after 10 weeks post challenge from one independent experiments. Arrow indicates inflammatory cells recruited area. Histology are representative of one of two mice from each group of one experiments. Scale Bars: 100 µm. (**D, E**) Parasite load of each *LmCen$^{-/-}$* immunized (Imm Chal) (n=5) & non-Immunized (Non-Imm chal) mice (n=5), ear (D) and dLN (E). Parasite burden was determined by limiting dilution assay. Results are represented as mean ± SEM. Data are representative of one experiment with five mice per group. Statistical analysis was performed by unpaired one-tailed t-test (*p -0.05; **p-0.009. (**F**) BALB/c mice were immunized intradermally into ear with $1 \times 10^7$ *LmCen$^{-/-}$* and, at 6 weeks post immunization, mice were challenged intradermally into alternate ear with 5,000 stationary phage FV9 *L. major* promastigotes. Representative photographs of ear lesion of immunized (n=5) and non-immunized mice (n=5) at 14 weeks post challenge infection Figure is representative of one experiment (**G**) BALB/c mice were immunized (n=5) subcutaneously in the footpad with 2 X $10^8$ *LmCen$^{-/-}$* parasites; six weeks post immunization challenged intradermally into the ear with 10,000 LV39 *L. major* parasites Representative photographs of ear lesion of immunized and non-immunized (n=5) mice at 10 weeks post challenge infection. Figure is representative of one of two independent experiments.

SUPPLEMENTARY FIGURE 4

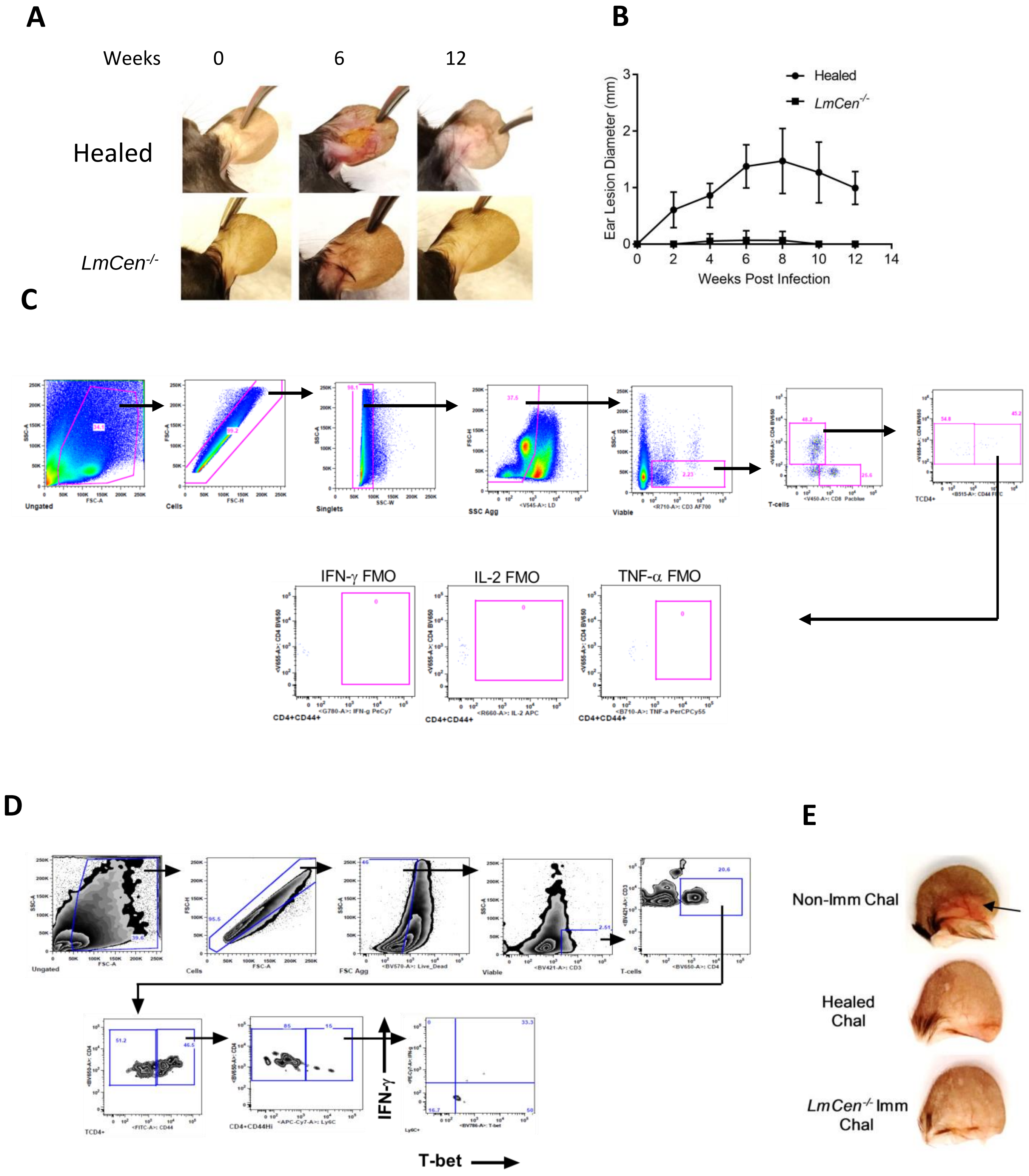

**SUPPLEMENTARY FIGURE 4**

**Immunization or leishmanization confer comparable host protection against *L. major* WT infection.**

**(A)** Photograph of representative ear showing the course of lesion development and subsequent cure from primary *LmWT* infection in healed group of mice. Results are representative of one of two independent experiments. (**B**) Ear lesion size was measured weekly for both *LmCen*$^{-/-}$ immunized (*LmCen*$^{-/-}$) (n=6) and healed from primary infected group (Healed) (n=6) of mice after intradermal inoculation of parasites. Results are represented as SD of 1 ear, 6 mice per group from two independent experiments. **(C)** The common gating strategies to determine positivity for each antibody (IL-2, TNF-α, IFN-γ) against intra cellular cytokines using fluorescence minus one (FMO) controls during comparative immune response between leishmanized (n=3) and *LmCen*$^{-/-}$ immunized mice (n=3). Results are representative of one experiment. (**D)** Common gating steps and representative zebra plots to determine positivity for IFN-γ antibody against intra cellular cytokines using fluorescence minus one (FMO) controls during 20h post challenge immune response in the ear of age matched non-immunized (n=6), healed (n=6) and *LmCen*$^{-/-}$ immunized mice (n=6) following needle challenge with wild type *L. major*-parasites. In the IFN-γ FMO of 20h post-challenge experiments, only two $CD4^{+}CD44^{Hi}$T-bet$^{+}$Ly-6C$^{+}$ -T cells are positive for IFN-γ (33.3%). Results are representative of one of two independent experiments (**D**) Representative photographs of ear lesion of age matched non-immunized (n=8), healed (n=5) and *LmCen*$^{-/-}$ immunized (n=6) mice at 5 weeks post *LmWT* infected sand fly challenge. Only age matched non immunized group develop lesion (black arrow) at 5 weeks post challenge compared to healed and *LmCen*$^{-/-}$ immunized mice. Pictures are representative of one experiment.
