# Supplementary material for: A second generation leishmanization vaccine with a markerless attenuated Leishmania major strain using CRISPR gene editing
Source: Nat Commun. 2020 Jul 10;11:3461. doi: 10.1038/s41467-020-17154-z (PMC7351751; doi:10.1038/s41467-020-17154-z)
Supplement: Supplementary file 2 — Reporting Summary [file 41467_2020_17154_MOESM2_ESM.pdf]

nature research

Corresponding author(s): Greg Matlashewski

Last updated by author(s): Jun 8, 2020

Reporting Summary

Nature Research wishes to improve the reproducibility of the work that we publish. This form provides structure for consistency and transparency in reporting. For further information on Nature Research policies, see Authors & Referees and the Editorial Policy Checklist .

Statistics

For all statistical analyses, confirm that the following items are present in the figure legend, table legend, main text, or Methods section.

n/a	Confirmed	
☐	☒	The exact sample size (*n*) for each experimental group/condition, given as a discrete number and unit of measurement
☐	☒	A statement on whether measurements were taken from distinct samples or whether the same sample was measured repeatedly
☐	☒	The statistical test(s) used AND whether they are one- or two-sided<br>*Only common tests should be described solely by name; describe more complex techniques in the Methods section.*
☒	☐	A description of all covariates tested
☒	☐	A description of any assumptions or corrections, such as tests of normality and adjustment for multiple comparisons
☐	☒	A full description of the statistical parameters including central tendency (e.g. means) or other basic estimates (e.g. regression coefficient) AND variation (e.g. standard deviation) or associated estimates of uncertainty (e.g. confidence intervals)
☒	☐	For null hypothesis testing, the test statistic (e.g. *F*, *t*, *r*) with confidence intervals, effect sizes, degrees of freedom and *P* value noted<br>*Give P values as exact values whenever suitable.*
☒	☐	For Bayesian analysis, information on the choice of priors and Markov chain Monte Carlo settings
☒	☐	For hierarchical and complex designs, identification of the appropriate level for tests and full reporting of outcomes
☒	☐	Estimates of effect sizes (e.g. Cohen's *d*, Pearson's *r*), indicating how they were calculated

Our web collection on statistics for biologists contains articles on many of the points above.

Software and code

Policy information about availability of computer code

Data collection	Illumina MiSeq (v2.6.2.1) was used to generate DNA sequence reads.
Data analysis	The Burrows-Wheeler aligner version 0.7.15 r1144 was used to align Illumina sequencing reads to the Leishmania Major Friedlin strain genome version 2016-05-28 (From tritrypdb.org). Samtools version 1.5-2 was used for file conversions and bedtools version 2.27.1 was used for coverage analysis. GraphPad Prism version 7.0 was used to generate graphs. Bio-Rad CFX Manager 3.1 for RT-PCR data analysis

For manuscripts utilizing custom algorithms or software that are central to the research but not yet described in published literature, software must be made available to editors/reviewers. We strongly encourage code deposition in a community repository (e.g. GitHub). See the Nature Research guidelines for submitting code & software for further information.

Data

Policy information about availability of data

All manuscripts must include a data availability statement. This statement should provide the following information, where applicable:

- Accession codes, unique identifiers, or web links for publicly available datasets
- A list of figures that have associated raw data
- A description of any restrictions on data availability

The data that support the findings of this study are available from the corresponding author upon reasonable request.

Field-specific reporting

Please select the one below that is the best fit for your research. If you are not sure, read the appropriate sections before making your selection.

Life sciences study design

All studies must disclose on these points even when the disclosure is negative.

Sample size	In all experiments we have used at least 3 animals per group to perform student t test analysis; no sample size calculation was necessary or performed since this study is of pre-clinical development stage.
Data exclusions	There was no data exclusions in this manuscript.
Replication	Where possible, all the experiments were replicated 2-4 times using monocytes obtained from independent blood donors, different animal models, and methods of challenge. We confirm that the attempts to replicate the findings are successful.
Randomization	In all experiments animals were randomly allocated between experimental groups
Blinding	Since this study is of pre-clinical development stage, blinding was not necessary or used in this study.

Reporting for specific materials, systems and methods

We require information from authors about some types of materials, experimental systems and methods used in many studies. Here, indicate whether each material, system or method listed is relevant to your study. If you are not sure if a list item applies to your research, read the appropriate section before selecting a response.

Materials & experimental systems

n/a	Involved in the study
☐	☒ Antibodies
☐	☒ Eukaryotic cell lines
☒	☐ Palaeontology
☐	☒ Animals and other organisms
☐	☒ Human research participants
☒	☐ Clinical data

Methods

n/a	Involved in the study
☒	☐ ChIP-seq
☐	☒ Flow cytometry
☒	☐ MRI-based neuroimaging

Antibodies

Antibodies used	CD16/CD32 (BD BioSciences, Cat: 553142, Clone: 2.4G2); AF700-CD3 (BD BioSciences, Cat: 557984, Clone: 500A2); BV421-CD3 (BD BioSciences, Cat: 562600, Clone: 145-2C11); BV650-CD4(Biolegend, Cat: 100555, Clone: RM4-5); Pacific Blue--CD8a (Biolegend, Cat: 100725, Clone: 53-6.7); FITC-CD44 (BD BioSciences, Cat: 553133, Clone: IM7); APC-Cy7-Ly6C (BD BioSciences, Cat: 560596, Clone: AL-21); APC-IL-2 (BD BioSciences, Cat: 554429, Clone: JES6-5H4); PE-Cyanine7-IFN-γ (Biolegend, Cat: 505826, Clone: XMG1.2); PerCP-Cyanine5.5-TNF-α (Biolegend, Cat: 506322, Clone: MP6-XT22); BV786-T-bet (BD BioSciences, Cat: 564141, Clone: Clone O4-46).
Validation	The antibodies have been titrated in our laboratory and described in previous publications:<br>Front Immunol. 2018. PMID: 29915577; Front Immunol. 2017. PMID:29312315; Scientific Reports, 2019. PMID: 31086209; Nat Commun. 2017. PMID: 29170498; Cell Metab. 2017. PMID: 28877454; Immunity. 2017. PMID: 29166589; Nat Commun. 2018. PMID: 29670099; Elife. 2017. PMID: 28895840; Cell. 2017. PMID: 28552348; Immunity. 2019. PMID: 30926234; Eur J Immunol. 2013. PMID: 24030473

Eukaryotic cell lines

Policy information about cell lines

Cell line source(s)	L. major Friedlin (FV9), L. major LV39 and L. major WR2855 strain; The WR2885 strain was isolated in August 29, 2008 from a lesion on the right upper arm of a US soldier at the Walter Reed hospital in Bethesda, MD.
Authentication	L. major Friedlin (FV9) and L. major LV39 confirmed to be L. major through genome sequening. WR2855 strain was acquired in Iraq and the cloned line was identified as L. major by polymerase chain reaction (PCR) and by isozyme assessment by a College of American Pathologists certified laboratory
Mycoplasma contamination	Not tested
Commonly misidentified lines (See ICLAC register)	No misidentified cell lines used

Animals and other organisms

Policy information about studies involving animals; ARRIVE guidelines recommended for reporting animal research

Laboratory animals	Female 6-8 weeks old mice are used in our studies; C57BL/6 and BALB/c mice; STAT-1KO (BALB/c); IFNg KO and RAG-2KO (C57BL/6). Female Lutzomyia longipalpis (Jacobina strain) are reared at the Laboratory of Malaria and Vector Research, NIAID/ NIH.
Wild animals	No wild animals were used in this study
Field-collected samples	No field collected samples were used in this study
Ethics oversight	The animal protocol for this study has been approved by the Institutional Animal Care and Use Committee at the Center for Biologics Evaluation and Research, US FDA (ASP 1995#26). The animal protocol is in full accordance with "The guide for the care and use of animals as descried in the US Public Health Service policy on Humane Care and Use of Laboratory Animals 2015" . The use of blood components (elutriated monocytes) from the Department of Transfusion Medicine, NIH was approved by the institutional Research Involving Human Subjects Committee (RIHSC#03-120B) at the US FDA. All animal studies at Ohio State University were performed in accordance with NIH guidelines for the humane care and use of animals and were approved by OSU IACUC. Animal experimental procedures performed at the National Institute of Allergy and Infectious Diseases (NIAID) were reviewed by the NIAID Animal Care and Use Committee under animal protocol LMVR4E. The NIAID DIR Animal Care and Use Program complies with the Guide for the Care and Use of Laboratory Animals and with the NIH Office of Animal Care and Use and Animal Research Advisory Committee guidelines. The housing condition of animals were followed standard guidelines by NIH guidelines for the humane care and use of animals. Animal experimental procedures performed at Nagasaki University were approved by the Institutional Animal Research Committee of Nagasaki University (No.1606211317 and 1505181227), the Nagasaki University Recombinant DNA Experiments Safety Committee (No. 1403041262 and 1407221278), and performed according to Japanese law for the Humane Treatment and Management of Animals.

Note that full information on the approval of the study protocol must also be provided in the manuscript.

Human research participants

Policy information about studies involving human research participants

Population characteristics	Elutriated monocytes from voluntary blood donors at the Department of Transfusion Medicine, NIH were used in this study. The anonymous donors that meet the blood donation criteria and CMV negative samples were used. We had no role in defining the donor inclusion criteria.
Recruitment	Since the monocytes from blood were isolated from voluntary blood donors at the NIH, we did not have any role in recruitment.
Ethics oversight	An institutional committee at the US FDA (Research Involving Human Subjects Committee, RIHSC) reviewed the protocol and approved the study prior to the start of the experiments. The study was reviewed biannually by the RIHSC.

Note that full information on the approval of the study protocol must also be provided in the manuscript.

Flow Cytometry

Plots

Confirm that:

- [x] The axis labels state the marker and fluorochrome used (e.g. CD4-FITC).
- [x] The axis scales are clearly visible. Include numbers along axes only for bottom left plot of group (a 'group' is an analysis of identical markers).
- [x] All plots are contour plots with outliers or pseudocolor plots.
- [x] A numerical value for number of cells or percentage (with statistics) is provided.

Methodology

Sample preparation	Please see Materials and methods and sections there of.
Instrument	Symphony (BD Biosciences, USA) analyzer equipped with 350, 405, 445, 488, 561, 638 and 785 nm LASER lines using DIVA software (v8).
Software	Cells were acquired on Symphony (BD Biosciences, USA) analyzer equipped with 350, 405, 445, 488, 561, 638 and 785 nm LASER lines using DIVA software (v8). Data were analyzed with the FlowJo software version 9.9.6 (BD, San Jose CA).
Cell population abundance	Please see Materials and methods and sections there of.
Gating strategy	For analysis, first doublets were removed using width parameter; dead cells were excluded based on staining with the Live/Dead Aqua dye. Lymphocytes were identified according to their light-scattering properties. CD4+ T-cells were identified as CD3+

lymphocytes uniquely expressing CD4. Upon further gating intracellular cytokines were measured. Gating strategy for the flow-cytometry analysis is described in the figures 6B, 6E and Supplementary figures 4C and 4D.

☒ Tick this box to confirm that a figure exemplifying the gating strategy is provided in the Supplementary Information.
